# Supplementary material for: Molecular interactome of HNRNPU reveals regulatory networks in neuronal differentiation and DNA methylation
Source: Nucleic Acids Res. 2026 Feb 12;54(4):gkag107. doi: 10.1093/nar/gkag107 (PMC12895067; doi:10.1093/nar/gkag107)
Supplement: gkag107_Supplemental_Files [file gkag107_supplemental_files.zip › Supplementary_File_1_15012026.pdf]

Molecular Interactome of HNRNPU Reveals Regulatory Networks in Neuronal Differentiation and DNA Methylation

Oksanen et al.

Supplementary Material

Content

Figures S1-S5

Tables S1-S12

Supplementary Figures

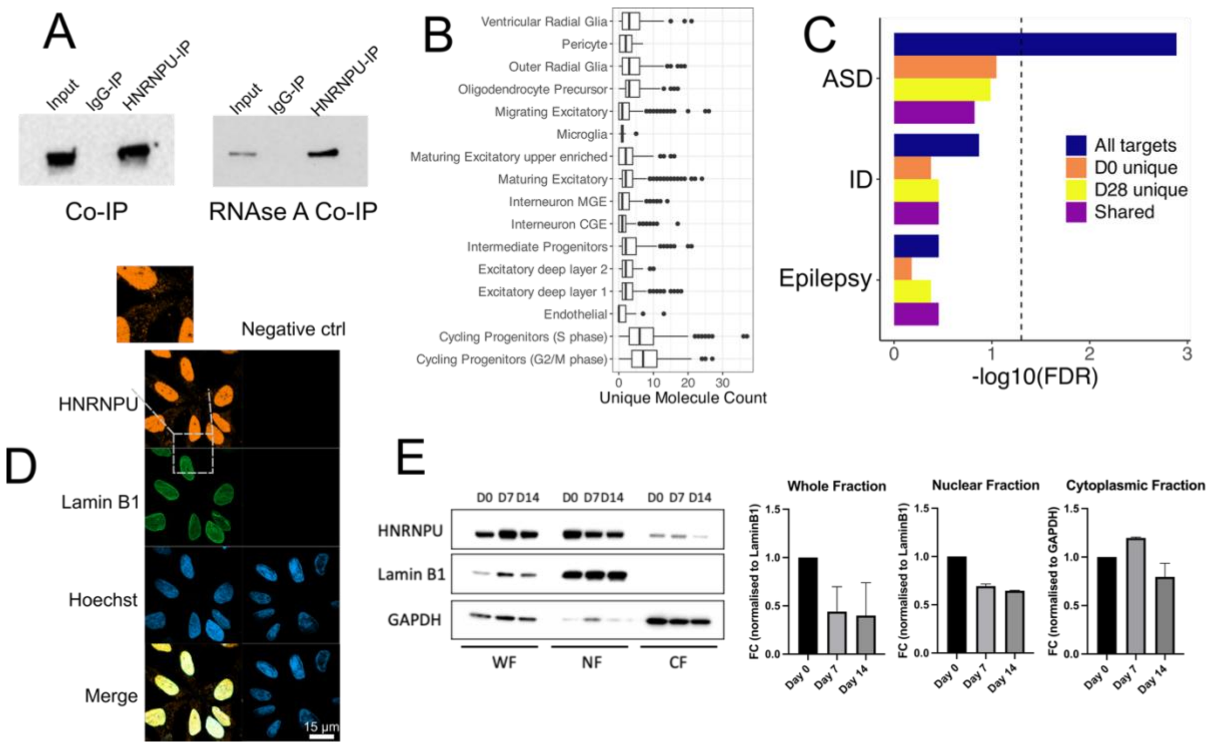

Figure S1: Characterization of HNRNPU protein-protein interactions and cytoplasmic localization A) Western blot analysis of untreated input sample (Input) and after Co-IP with either mock IgG control (IgG) or HNRNPU (IP). Same setting was used for Co-IP after RNase A treatment (right). B) *HNRNPU* mRNA expression per cell type in the scRNA-seq reference data set used for cell type enrichment analysis. C) Enrichment of the PPI partners of HNRNPU at D0 and D28 in autism (ASD), intellectual disability (ID) and epilepsy risk gene lists. The vertical dotted line represents the significance threshold of FDR<0.05 (-log10(FDR)>1.3) after hypergeometric analysis. D) Immunocytochemistry staining of HNRNPU, Lamin B1 and Hoechst in $CTRL_{Male}$ cells fixed with methanol, imaged with super resolution microscope. Scale bar 15 µm. E) Western blot and densitometry quantification after subcellular fractionation of $CTRL_{Male}$ cells after 0, 7 and 14 days in differentiation. WF, whole-cell fraction; NF, nuclear fraction; CF, cytoplasmic fraction. Represented are HNRNPU, nuclear marker Lamin B1 and cytoplasmic marker GAPDH. Whole-cell fraction and nuclear fraction were normalized to Lamin B1 expression and cytoplasmic fraction to GAPDH expression. (n=2)

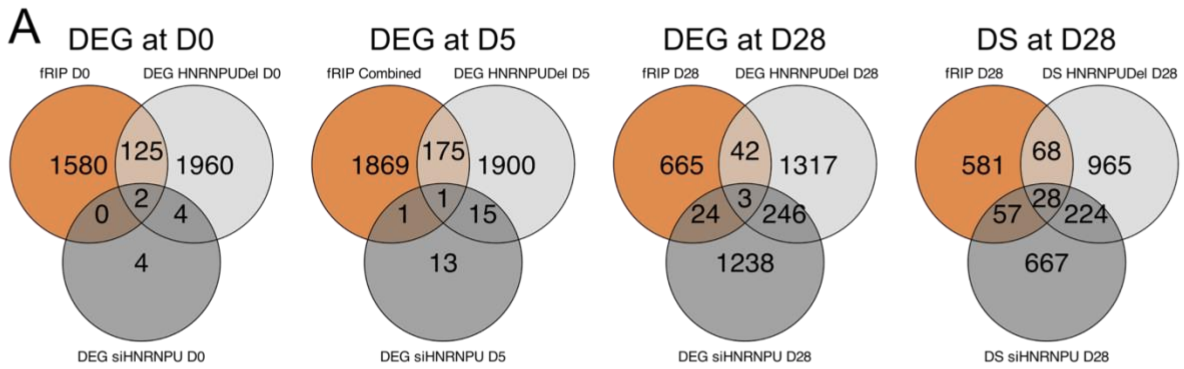

Figure S2: Overlap of HNRNPU RNA targets with differential expression and splicing in HNRNPU deficiency A) Venn diagrams representing overlaps between HNRNPU targets that are simultaneously differentially expressed (DE) at D0, D5 and D28, or differentially spliced (DS) at D28 in relation to *HNRNPU* deficiency. A combined list of HNRNPU targets at D0 and D28 was used when comparing with DE genes at D5, as fRIP-Seq was prepared only at D0 and D28. RNA-Seq data were from our earlier publication (Mastropasqua et al., 2023). siHNRNPU refers to siRNA silenced samples and HNRNPUdel refers to a patient cell line with heterozygous deletion of *HNRNPU*.

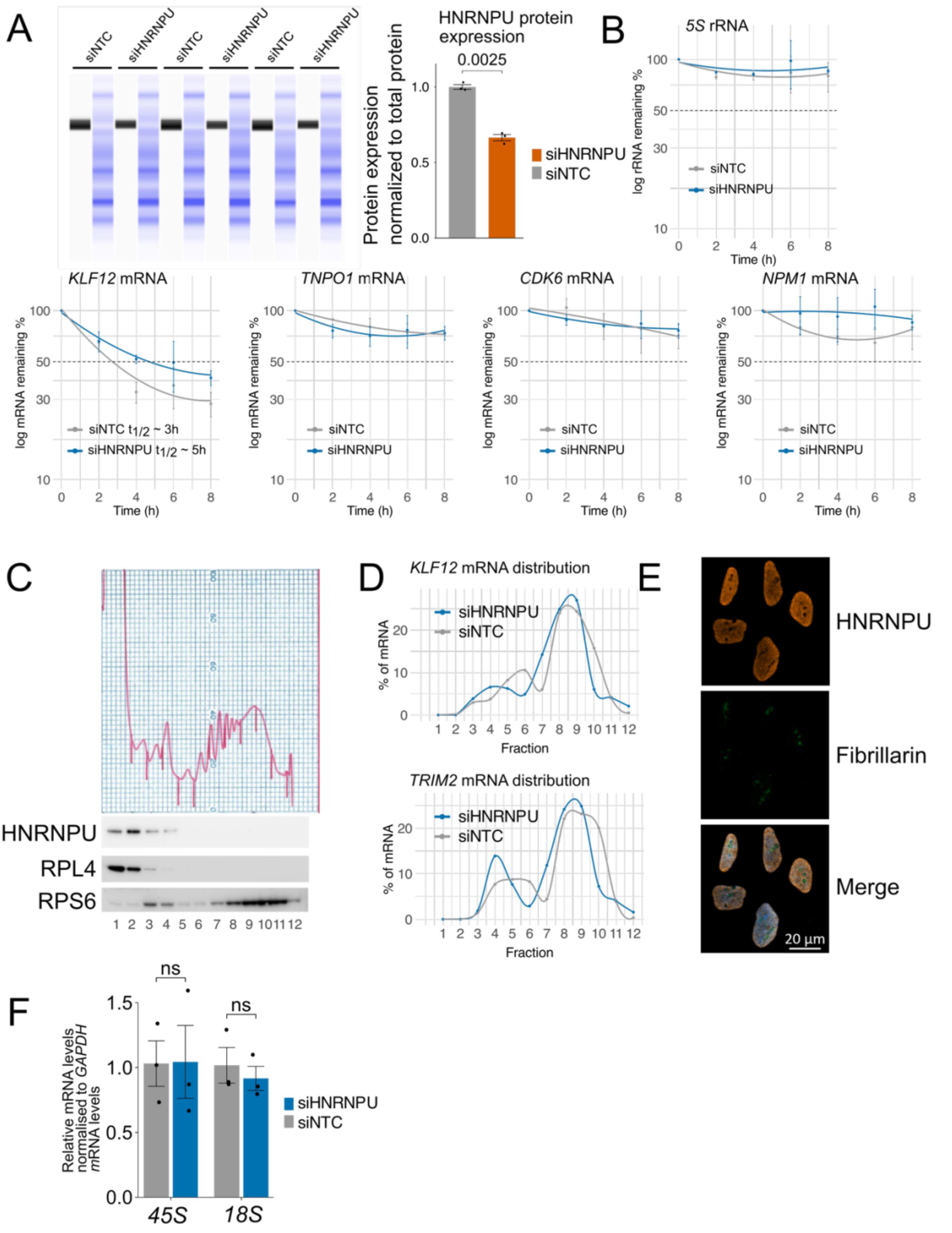

Figure S3: Impact of HNRNPU knockdown on mRNA stability and translation in neural cells. A) Capillary western blot and quantification of HNRNPU expression after siRNA silencing at D0. Data were normalized to total protein and represented as fold change relative to siNTC samples. Error bars represent SEM and statistical significance was calculated using Student's T-test followed by Benjamini-Hochberg multiple correction (n=3). B) RT-qPCR analysis of the

stability of a stable control *5S* rRNA, HNRNPU targets *KLF12*, *TNPO1* and *CDK6* mRNAs as well as a non-target *NPM1* mRNA at D0 after HNRNPU silencing and actinomycin D treatment for the indicated times (n=3). C) Polysome profiling at D0, followed by HNRNPU, RPL4 and RPS6 western blot analysis. Fractions 1-2 represent fractions without ribosomal material, 3-5 are 40S, 60S, 80S monosomes, 6-8 low-molecular-weight polysomes and 9-12 high-molecular-weight polysomes. D) Relative mRNA distribution of HNRNPU targets *KLF12* and *TRIM2* mRNAs on polysome gradients as assessed by RT-qPCR analysis (n=1). E) Immunocytochemistry staining of HNRNPU, nucleoli marker Fibrillarin and Hoechst in $CTRL_{Male}$ cells imaged with a confocal microscope. Scale bar 20 μm. F) Quantification of *45S* rRNA and *18S* rRNA levels after HNRNPU silencing measured by RT-qPCR. Error bars represent SEM and statistical significance was calculated using Student's T-test followed by Benjamini-Hochberg multiple correction (n=3).

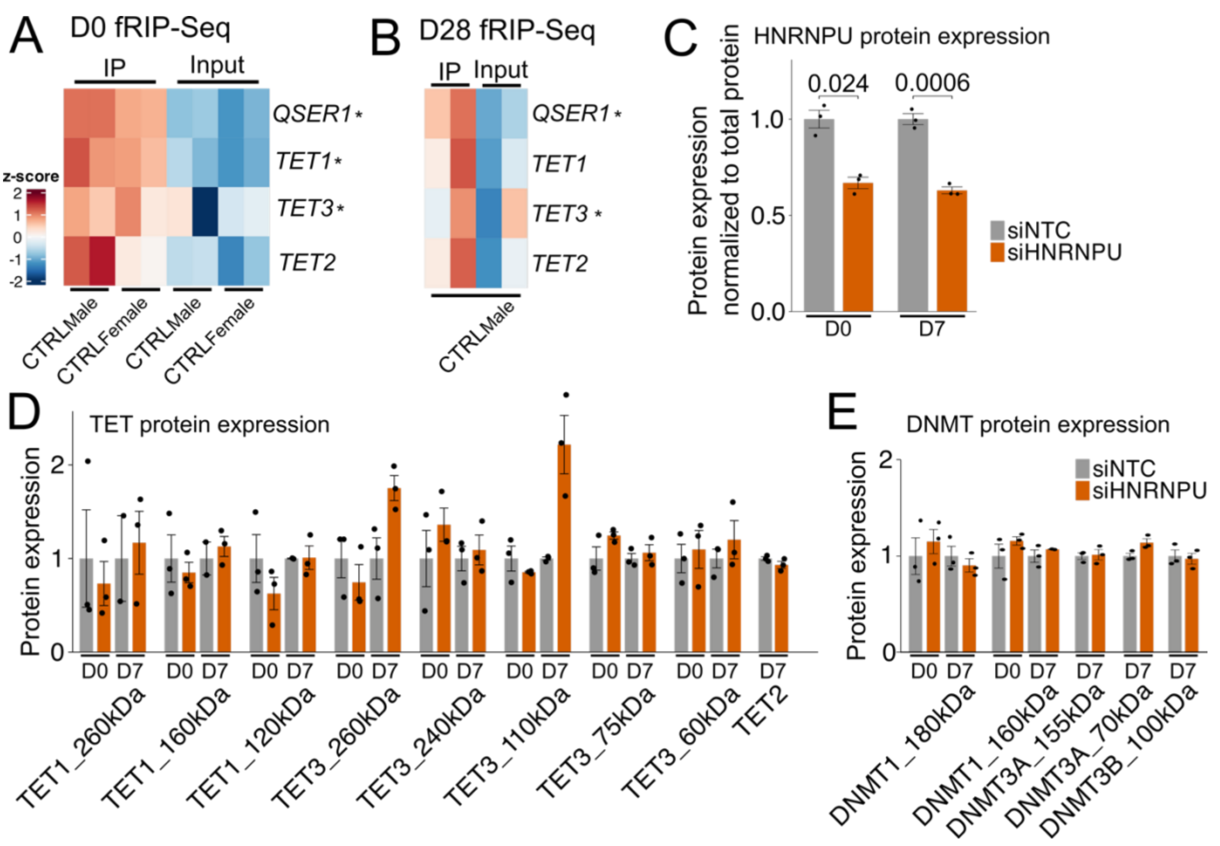

Figure S4: HNRNPU targets mRNAs encoding DNA methylation regulators in differentiating neural cells A-B) Heatmap of *QSER1*, *TET1*, *TET3* and *TET2* mRNAs from fRIP-Seq data from $CTRL_{Male}$ at D0 (A) and D28 (B). Asterisks represent significant HNRNPU targets. *TET3* mRNA was a significant HNRNPU target based on CLAM peak calling analysis at D28. C) Quantification of HNRNPU expression after silencing at D0 and D7 measured by capillary western blot. Data were normalized to total protein and represented as fold change relative to siNTC samples. Error bars represent SEM, and statistical significance was calculated using Student's t-test followed by Benjamini-Hochberg multiple correction (n=3). D-E) Western blot and capillary western blot quantification of TET1/2/3 (D) and DNMT1/3A/3B (E). Data were normalized to total protein for capillary western blot and either Lamin B1 or GAPDH for western blot and represented as fold change relative to siNTC samples. Western blots and images of capillary western blots are presented in Figure S5. Error bars represent SEM, and statistical significance was calculated using Student's t-test followed by Benjamini-Hochberg multiple correction (n=3).

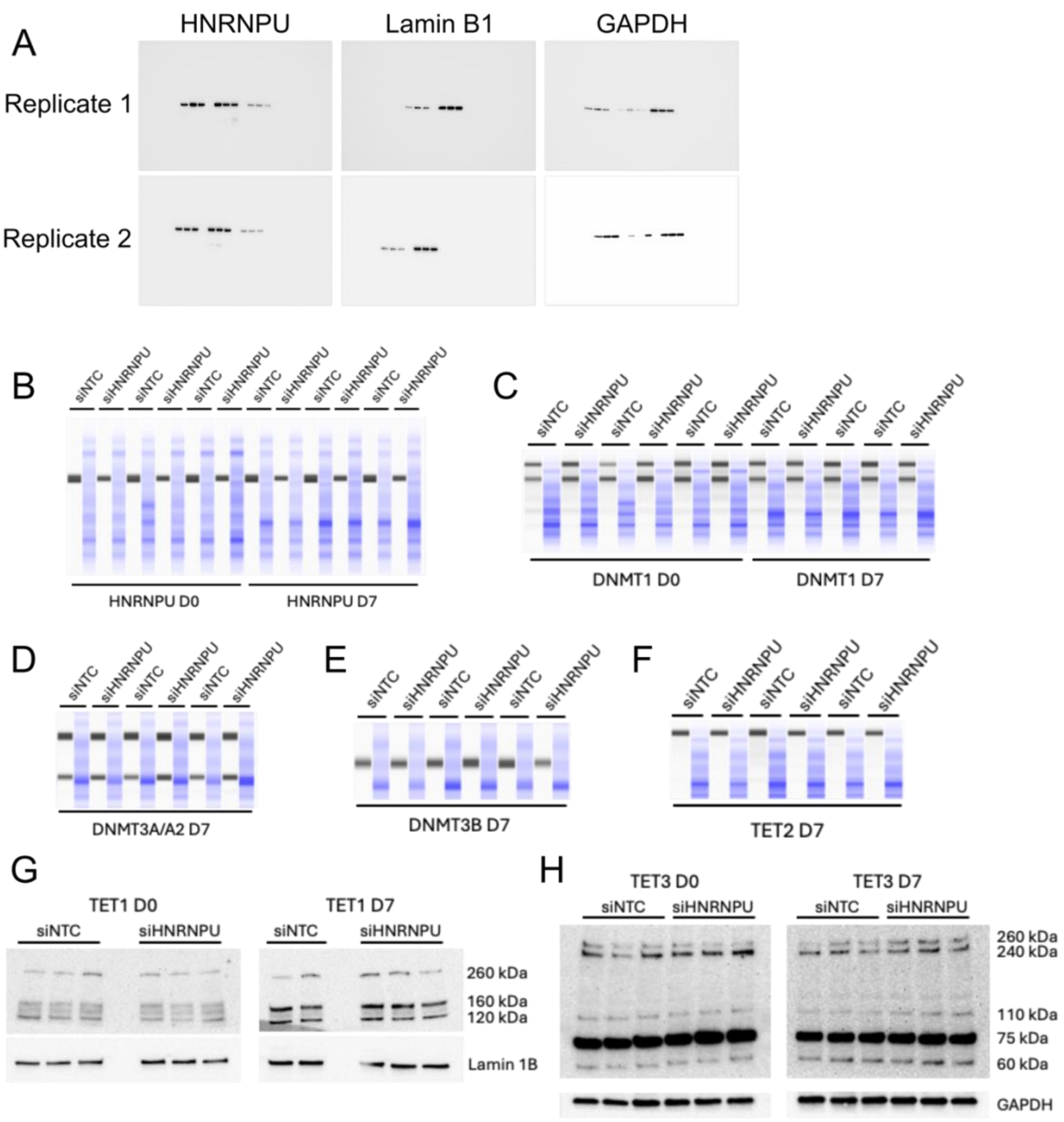

Figure S5: Full western blot and capillary western blot images. A) Full images of western blots related to figure S1. Represented are HNRNPU, nuclear marker Lamin B1 and cytoplasmic marker GAPDH, from two biological replicates. B-H) Capillary western blot images representing HNRNPU (B), DNMT1 (C), DNMT3A/A2 (D), DMT3B (E), and TET2 (F) either at both D0 and D7 or only D7 time points. G-F) Full images of western blots of TET1 (G) and TET3 (F) at D0 and D7 time points.

Supplementary Tables

Table S1. A list of motifs compiled from MEME-Suite (v.5.5.7) and RBPMap (v.1.2), used in motif enrichment analysis of fRIP-Seq data. See separate file.

Table S2. Primers used in RT-qPCR analysis

mRNA	Forward	Reverse
5S	CGCCCGATCTCGTCTGAT	GGTCTCCCATCCAAGTACTAACCA
CDK6	GGCTCTAACCTCAGTGGTCGT	CAACACTCCAGAGATCCACGG
ERBB4	TAGTTCAGGATGTGGACGTTGC	ACACACCGTCCTTGTCAAAGT
GAPDH	AAGGTGAAGGTCGGAGTCAAC	GGGGTCATTGATGGCAACAATA
HNRNPU	AGTTTAACAGAGGTGGTGGCC	GCCCCTCCTATTATATCCGCC
KLF12	CAGTCGGTGCCTGTTGTCTA	GGTCCATTTGTGCTTTGCCA
NPM1	GCTGGTGCAAAGGATGAGTTG	CCAAGGGAAACCGTTGGCT
MYC	TTCTCTCCGTCCTCGGATTCTCTG	TCTTCTTGTTCCTCCTCAGAGTCG
QSER1	CACCTCAGCCTACTGGTCTT	CCTGAGCAGTTCGGGATTCA
TNPO1	GGCCTGACCTCTTACCAAAAC	ACCAAATGCTCCCTCACAG
TRIM2	TGAAGGCACCAACATCCCAA	GTAGTTCTGCAGGCACCTCTC
45S	TCGTGCTGCCCTCTCGG	GGAACGACACACCACCGTT
18S	CGAACGTCTGCCCTATCAACTT	ACCCGTGGTCACCATGGTA

Table S3. List of significantly enriched HNRNPU protein-protein interacting partners at D0 and D28. Column ‘Interaction_Type’ specifies whether the interaction was classified direct or RNA-assisted based on RNAse A treated samples, while value ‘direct, RNA-assisted’ indicates that the interaction was direct at D0 and RNA-assisted at D28. Column ‘Time_Point’ specifies whether the protein was interacting with HNRNPU at D0, D28 or shared between both time points. Column ‘Biogrid’ specifies wether the interaction with HNRNPU had been deposited to BioGrid database. Listed are peak areas, average peak areas, fold change, P values and adjusted P values for D0 and D28 samples, with and without RNAse A treatment. See separate file.

Table S4. Results of AlphaPulldown analysis. Significance was determined by ipTM > 0.6. Column 'HNRNPU Domain' specifies which functional domain of HNRNPU was involved in the interaction.

HNRNPU Interactor	HNRNPU Domain	Inter-face	Num_intf residues	Polar	Hydro-phobhic	Charged	Contact pairs	sc	hb	sb	int_solv_en	int_area	pi_score	ipTM	mpDockQ /pDockQ
DDX39A	SAP	C_B	9	0.111	0.333	0.444	8	0.616	5	3	-0.91	461.31	1.04	0,8601	0,2004
DDX17	SPRY	C_B	22	0.045	0.409	0.318	25	0.405	5	0	-10.26	1312.03	-0.02	0,8482	0,3517
DDX5	SPRY	C_B	22	0.091	0.409	0.273	19	0.411	3	1	-9.18	1123.81	-0.36	0,8397	0,3226
FBL	SAP	C_B	11	0.364	0.273	0.273	11	0.578	3	4	-0.18	468.91	-0.11	0,8135	0,1860
RPS4Y2	SAP	C_B	10	0.2	0.3	0.5	8	0.446	2	1	-4.4	569.87	-0.28	0,7940	0,1490
EIF3L	SAP	C_B	9	0.333	0.333	0.222	7	0.642	5	4	-0.59	479.7	0.63	0,7911	0,1344
EIF3C	SPRY	C_B	17	0.294	0.588	0.118	16	0.501	1	0	-7.32	547.05	-0.64	0,7809	0,1271
RPS4X	SAP	C_B	10	0.2	0.3	0.5	8	0.435	3	0	-4.4	546.81	-0.26	0,7791	0,1420
HNRNPU	ATPase	C_B	29	0.448	0.172	0.241	29	0.519	16	4	-5.25	1237.71	0.51	0,7646	0,2270
GIGYF2	ATPase	C_B	25	0.32	0.28	0.24	24	0.437	9	6	-6.6	1069.05	-0.82	0,7614	0,1619
SRSF2	SAP	C_B	9	0.111	0.222	0.444	9	0.504	5	2	-0.2	345.37	-0.19	0,7598	0,1467
FAU	SPRY	C_B	41	0.22	0.39	0.146	57	0.67	14	3	-13.84	1412.91	2.22	0,7225	0,4704
USF1	SPRY	C_B	27	0.259	0.481	0.185	31	0.582	8	0	-14.39	1177.5	1.28	0,7157	0,1324
EIF5B	ATPase	C_B	24	0.125	0.458	0.25	19	0.359	1	7	-11.23	1155.07	-0.48	0,7149	0,1581
SLIRP	SAP	C_B	15	0.133	0.467	0.2	15	0.526	1	0	-7.89	487.12	0.02	0,7148	0,1936
RPL23A	SAP	C_B	9	0.222	0.222	0.333	7	0.475	1	2	-0.56	260.88	-1.22	0,7120	0,0759
SMARCB1	ATPase	C_B	17	0.353	0.353	0.118	12	0.456	7	4	-8.68	962.87	-0.68	0,7056	0,2090
EFTUD2	ATPase	C_B	12	0.333	0.417	0.083	9	0.434	3	4	-1.92	590.94	-1.57	0,7020	0,0987
EIF2A	SPRY	C_B	7	0.143	0.429	0.143	4	0.626	2	0	-3.17	712.39	-0.53	0,6991	0,1037
PDAP1	SPRY	C_B	20	0.3	0.25	0.35	23	0.425	6	4	1.77	683.2	-1.6	0,6955	0,0876
CCDC136	ATPase	C_B	24	0.292	0.292	0.25	18	0.441	5	9	-9.57	1392.82	-0.73	0,6856	0,1636
SRSF10	SAP	C_B	11	0.182	0.182	0.545	11	0.613	3	2	-0.81	355.87	0.68	0,6691	0,1443
USF2	SPRY	C_B	46	0.239	0.413	0.196	51	0.443	12	0	-24.66	1768.75	0.75	0,6671	0,1560
RPL31	ATPase	C_B	7	0.143	0.571	0.286	5	0.628	2	5	0.74	341.16	-0.2	0,6599	0,0369

RIOK2	SPRY	C_B	87	0.322	0.356	0.195	173	0.076	22	1	-17.72	2274.53	0.79	0,6497	0,5619
FUBP3	SPRY	C_B	70	0.2	0.357	0.243	106	0.268	14	10	-10.29	2143.99	0.05	0,6493	0,2939
RTN1	ATPase	C_B	34	0.235	0.441	0.206	28	0.305	7	2	-13.91	1331.93	-1.27	0,6492	0,2409
DAP3	ATPase	C_B	11	0.455	0.364	0.091	12	0.579	4	1	0.87	358.9	-0.43	0,6436	0,0762
SMARCC2	SPRY	C_B	35	0.257	0.457	0.171	39	0.476	7	0	-14.87	1287.24	0.29	0,6434	0,0951
LASP1	SPRY	C_B	28	0.286	0.464	0.25	34	0.19	5	5	-17.4	1224.31	-0.42	0,6419	0,0813
SLTM	ATPase	C_B	37	0.324	0.324	0.27	40	0.257	15	5	-8.85	1395.69	-1.11	0,6087	0,1657
JPT2	SPRY	C_B	19	0.158	0.579	0.105	24	0.583	8	2	-11.88	795.78	1.31	0,6007	0,1870

Table S5. GO biological process enrichment analysis of HNRNPU PPIs and mRNA targets at D0 and D28 and promoter DMRs that overlapped H3K4me3 CUT&RUN peaks at D0 and D7. The input list for PPIs consisted of both direct and RNA-assisted PPIs. The PPI and fRIP-Seq input lists were split by all, D0-unique, D28-unique and shared PPIs/RNA targets, while DMRs were split by all, hyper- and hypomethylated DMRs. Sheet 1: HNRNPU PPI enrichment all proteins. Sheet 2: HNRNPU PPI enrichment at D0-unique. Sheet 3: HNRNPU PPI enrichment at D28-unique. Sheet 4: HNNRPU PPI enrichment shared proteins. Sheet 5: HNRNPU RNA target enrichment all targets. Sheet 6: HNRNPU RNA target enrichment D0-unique. Sheet 7: HNRNPU RNA target enrichment D28-unique. Sheet 8: HNRNPU RNA target enrichment shared proteins. Sheet 9: All promoter DMRs overlapping with H3K4me3 enrichment at D0. Sheet 10: Hypomethylated promoter DMRs overlapping with H3K4me3 enrichment at D0. Sheet 11: Hypermethylated promoter DMRs overlapping with H3K4me3 enrichment at D0. Sheet 12: All promoter DMRs overlapping with H3K4me3 enrichment at D7. Sheet 13: Hypomethylated promoter DMRs overlapping with H3K4me3 enrichment at D7. Sheet 14: Hypermethylated promoter DMRs overlapping with H3K4me3 enrichment at D7. See separate file.

Table S6. Cell type enrichment analysis (EWCE) of HNRNPU PPIs (IP-MS) and RNA targets (fRIP-Seq) at D0 and D28. The input lists were separated for all proteins/targets, D0-unique, D28-unique and shared proteins/targets. See separate file.

Table S7. Enrichment analysis of HNRNPU PPIs (IP-MS) and HNRNPU mRNA targets (fRIP-Seq) on autism (ASD), intellectual disability (ID) and epilepsy risk gene lists (hypergeometric test). Background gene lists were obtained from our previous RNA-Seq data from Mastropasqua et al., 2023, with BaseMean > 20. Q refers to intersection of PPI and NDD risk genes, and m refers to a number of NDD risk genes. The input lists were separated for all proteins/targets, D0-unique, D28-unique and shared proteins/targets.

Assay	Input	NDD	Background	q	m	P-value	FDR	-log10(P-value)
IP-MS	All proteins	SFARI	19679	29	964	0,00010786	0,00129436	2,8879
IP-MS	All proteins	ID	19679	34	1785	0,04511947	0,13535841	0,8685
IP-MS	All proteins	Epilepsy	19679	13	749	0,26242334	0,34989779	0,4561
IP-MS	D0 unique	SFARI	15987	19	883	0,014969	0,089814	1,0467
IP-MS	D0 unique	ID	15987	23	1703	0,3810244	0,41947198	0,3773
IP-MS	D0 unique	Epilepsy	15987	8	705	0,66096173	0,66096173	0,1798
IP-MS	D28 unique	SFARI	19162	5	958	0,0257849	0,1031396	0,9866
IP-MS	D28 unique	ID	19162	5	1777	0,20333734	0,34989779	0,4561
IP-MS	D28 unique	Epilepsy	19162	2	747	0,38451598	0,41947198	0,3773
IP-MS	Shared proteins	SFARI	19679	5	964	0,06270102	0,15048245	0,8225
IP-MS	Shared proteins	ID	19679	6	1785	0,20550076	0,34989779	0,4561
IP-MS	Shared proteins	Epilepsy	19679	3	749	0,23428524	0,34989779	0,4561
fRIP-Seq	All targets	SFARI	19679	212	964	2,6379E-27	1,5827E-26	25,8006
fRIP-Seq	All targets	ID	19679	376	1785	1,0225E-44	1,227E-43	42,9112
fRIP-Seq	All targets	Epilepsy	19679	145	749	6,0791E-14	1,2158E-13	12,9151
fRIP-Seq	D0 unique	SFARI	15987	115	883	3,4952E-07	5,2427E-07	6,2804
fRIP-Seq	D0 unique	ID	15987	232	1703	6,7988E-16	1,6317E-15	14,7874
fRIP-Seq	D0 unique	Epilepsy	15987	84	705	0,00031282	0,00037538	3,4255

fRIP-Seq	D28 unique	SFARI	19162	34	958	9,3494E-05	0,00012466	3,9043
fRIP-Seq	D28 unique	ID	19162	48	1777	0,00205961	0,00224685	2,6484
fRIP-Seq	D28 unique	Epilepsy	19162	20	747	0,04393156	0,04393156	1,3572
fRIP-Seq	Shared targets	SFARI	19679	63	964	8,3903E-17	2,5171E-16	15,5991
fRIP-Seq	Shared targets	ID	19679	96	1785	1,1236E-19	4,4944E-19	18,3473
fRIP-Seq	Shared targets	Epilepsy	19679	41	749	6,3646E-09	1,0911E-08	7,9621

Table S8. List of significant HNRNPU RNA targets at D0 and D28. Sheet 1: HNRNPU mRNA targets based on CLAM peak calling from fRIP-Seq data at D0 and D28. The column 'Timepoint' specifies whether the mRNA was a target at D0, D28 or shared target at both time points. Sheet 2: HNRNPU mRNA targets from fRIP-Seq data that show either differential expression or differential splicing in HNRNPU deficient 2D (Mastropasqua et al. 2023), 3D (Ressler et al. 2023) or mouse (Sapir et al. 2022) datasets. See separate file.

Table S9. MEME enrichment analysis of HNRNPU RNA motif in CLAM peak regions at D0 and D28.

Time Point	CONSENSUS	TP	TP%	FP	FP%	ENR _RATIO	SCORE _THR	PVALUE	EVALUE	QVALUE
D0	UGUAUUG	9235	91.64	9320	92.48	0.991	1.5	8.06e-1	8.06e-1	8.06e-1
D28	UGUAUUG	18486	92.84	18575	93.29	0.995	1.4	8.49e-1	8.49e-1	8.49e-1

Table S10. MEME enrichment analysis results at D0 and D28. The motifs used were from Table S1 and the input was RNA sequences from CLAM peak regions, split by All targets, D0-unique, D28-unique and shared targets. The D28-unique analysis yielded no significant results.

Input	Motif ID	Protein	Concensus	TP	TP%	FP	FP%	Enr ratio	Score thr	P-value	E-value	Q-value
All targets	RNCMPT00160	HNRNPH2	GGGAGGG	2091	8.25	1447	5.71	1.44	8.9	1.11e-27	1.42e-25	7.78e-26
All targets	RBPMap-FUBP1-2	FUBP1	UDUUU	4344	17.14	3716	14.67	1.17	7.4	1.40e-12	1.79e-10	4.90e-11
All targets	RNCMPT00086	ZC3H14	UUUGUUU	1894	7.47	1501	5.92	1.26	8.4	8.19e-12	1.05e-9	1.91e-10
All targets	RNCMPT00090	SRSF10	AGAGAMA	373	1.47	217	0.86	1.72	10	6.91e-11	8.84e-9	1.21e-9
All targets	RNCMPT00041	MSI1	UAGUWRG	3723	14.69	3195	12.61	1.17	7.6	1.16e-10	1.48e-8	1.62e-9
All targets	RNCMPT00117	HuR	UUUGUUU	1459	5.76	1137	4.49	1.28	9.1	1.41e-10	1.81e-8	1.65e-9
All targets	RBPMap-ELAVL4-2	ELAVL4	UUWUU	6310	24.90	5633	22.23	1.12	6.2	3.06e-10	3.92e-8	3.06e-9
All targets	RNCMPT00112	HuR	UUUGUUU	1023	4.04	764	3.02	1.34	9.8	4.87e-10	6.23e-8	4.26e-9
All targets	RNCMPT00070	SNRNP70	RWUCAAG	1040	4.10	783	3.09	1.33	10	9.54e-10	1.22e-7	7.41e-9
All targets	RNCMPT00044	PCBP2	CCUYCCC	996	3.93	762	3.01	1.31	9.1	1.31e-8	1.68e-6	9.17e-8
All targets	RBPMap-BOLL-2	BOLL	UUUDUUU	937	3.70	713	2.81	1.31	7.2	1.92e-8	2.46e-6	1.22e-7
All targets	RNCMPT00089	SRSF10	AGAGARR	208	0.82	110	0.43	1.88	10	2.11e-8	2.70e-6	1.23e-7
All targets	RBPMap-ELAVL4-1	ELAVL4	UAAUU	2609	10.30	2240	8.84	1.16	9.4	6.21e-8	7.95e-6	3.34e-7
All targets	RNCMPT00088	SRSF10	AGAGARR	146	0.58	69	0.27	2.1	9.9	8.13e-8	1.04e-5	4.06e-7
All targets	RNCMPT00150	ESRP2	UGGGGAU	1346	5.31	1090	4.30	1.23	9	1.16e-7	1.49e-5	5.42e-7
All targets	RNCMPT00177	SFPQ	GURGUKU	439	1.73	300	1.18	1.46	9.2	1.78e-7	2.28e-5	7.79e-7
All targets	RBPMap-EIF4G2-3	EIF4G2	GGUYGC	1090	4.30	865	3.41	1.26	10	1.97e-7	2.53e-5	8.12e-7

All targets	RBPMap-EWSR1-2	EWSR1	GGGDGGGG	176	0.69	94	0.37	1.86	11	3.41e-7	4.37e-5	1.33e-6
All targets	RNCMPT00187	BRUNOL6	UGUGDKG	504	1.99	378	1.49	1.33	9.9	1.25e-5	1.59e-3	4.58e-5
All targets	RBPMap-FUBP3-1	FUBP3	UAUAU	2390	9.43	2127	8.39	1.12	8.5	4.82e-5	6.17e-3	1.69e-4
All targets	RNCMPT00050	RBM3	GAUACGA	3894	15.37	3561	14.05	1.09	7	6.01e-5	7.69e-3	2.00e-4
All targets	RNCMPT00019	SRSF10	AGAGAAA	211	0.83	140	0.55	1.5	10	8.91e-5	1.14e-2	2.83e-4
All targets	RNCMPT00067	SRSF9	GGRWGGA	208	0.82	138	0.54	1.5	11	9.92e-5	1.27e-2	3.02e-4
All targets	RBPMap-ESRP1-1	ESRP1	GGGKGG	705	2.78	576	2.27	1.22	9	1.72e-4	2.21e-2	5.02e-4
All targets	RNCMPT00113	RBM4	GCGCGSG	2927	11.55	2661	10.50	1.1	6.2	1.96e-4	2.51e-2	5.48e-4
All targets	RNCMPT00032	HuR	UUWUUUU	479	1.89	378	1.49	1.27	10	3.14e-4	4.02e-2	8.44e-4
All targets	RBPMap-FUS-1	FUS	GGKGG	806	3.18	675	2.66	1.19	10	3.62e-4	4.64e-2	9.32e-4
All targets	RNCMPT00162	LIN28A	CGGAGGR	6781	26.76	6393	25.23	1.06	6.1	3.73e-4	4.78e-2	9.32e-4
All targets	RNCMPT00154	RBM5	GAAGGAG	2366	9.34	2147	8.47	1.1	8.2	5.86e-4	7.50e-2	1.41e-3
All targets	RNCMPT00152	RBMS1	UAUAUAS	399	1.57	313	1.24	1.27	11	7.14e-4	9.14e-2	1.66e-3
All targets	RNCMPT00173	RBMS3	UAUAUAB	1061	4.19	919	3.63	1.15	9.5	7.62e-4	9.76e-2	1.72e-3
All targets	RNCMPT00026	HNRNPK	CCAAMCC	348	1.37	271	1.07	1.28	11	1.11e-3	1.43e-1	2.43e-3
All targets	RNCMPT00004	BRUNOL4	UGUGUGU	718	2.83	609	2.40	1.18	9.3	1.51e-3	1.93e-1	3.19e-3
All targets	RNCMPT00056	RBM8A	GCGCGCG	1057	4.17	929	3.67	1.14	9.4	2.18e-3	2.79e-1	4.49e-3
All targets	RNCMPT00013	DAZAP1	UAGGUAR	69	0.27	40	0.16	1.71	12	3.52e-3	4.51e-1	7.03e-3
All targets	RNCMPT00166	BRUNOL5	UGUGUGU	634	2.50	541	2.14	1.17	10	3.63e-3	4.64e-1	7.04e-3
All targets	RNCMPT00057	RBMS3	AUAUAUM	454	1.79	376	1.48	1.21	11	3.74e-3	4.79e-1	7.08e-3
All targets	RNCMPT00107	SRSF1	GGAGGAN	2603	10.27	2415	9.53	1.08	8.8	4.14e-3	5.30e-1	7.48e-3

All targets	RNCMPT00064	SART3	ARAAAAA	3557	14.04	3337	13.17	1.07	7.7	4.17e-3	5.34e-1	7.48e-3
All targets	RNCMPT00155	PABPC1	ARAAAAA	3556	14.03	3337	13.17	1.07	7.7	4.32e-3	5.53e-1	7.55e-3
All targets	RNCMPT00049	RBM28	GWGUAGW	226	0.89	175	0.69	1.29	11	6.22e-3	7.96e-1	1.06e-2
All targets	RBPMap-FUBP1-1	FUBP1	UAUGUAU	485	1.91	411	1.62	1.18	9.7	7.35e-3	9.40e-1	1.20e-2
All targets	RNCMPT00171	PABPC5	AGAAAAU	1231	4.86	1112	4.39	1.11	9	7.38e-3	9.45e-1	1.20e-2
All targets	RNCMPT00052	RBM4	GCGCGGG	4891	19.30	4656	18.37	1.05	3.7	8.31e-3	1.06e0	1.32e-2
All targets	RBPMap-DAZ3-1	DAZ3	ACGUUU	285	1.12	233	0.92	1.22	12	1.25e-2	1.60e0	1.94e-2
All targets	RBPMap-FUBP3-3	FUBP3	UUUWU	1164	4.59	1061	4.19	1.1	8	1.53e-2	1.96e0	2.25e-2
All targets	RNCMPT00156	CNOT4	GACAGAN	2386	9.42	2238	8.83	1.07	8.4	1.53e-2	1.96e0	2.25e-2
All targets	RNCMPT00186	PCBP1	CCUWWCC	3299	13.02	3125	12.33	1.06	8	1.54e-2	1.98e0	2.25e-2
All targets	RNCMPT00109	SRSF1	GGAGGRV	4572	18.04	4380	17.29	1.04	7.3	2.18e-2	2.78e0	3.08e-2
All targets	RNCMPT00106	SRSF1	GGAGGAM	3157	12.46	2998	11.83	1.05	8.3	2.20e-2	2.82e0	3.08e-2
All targets	RNCMPT00108	SRSF1	GGAGGRV	4046	15.97	3883	15.32	1.04	7.6	3.44e-2	4.41e0	4.72e-2
All targets	RNCMPT00116	YBX1	AACAUCA	648	2.56	584	2.30	1.11	11	3.63e-2	4.65e0	4.88e-2
D0-unique	RNCMPT00160	HNRNPH2	GGGAGGG	1007	9.35	719	6.68	1.4	8.6	2.20e-12	2.81e-10	1.76e-10
D0-unique	RNCMPT00088	SRSF10	AGAGARR	45	0.42	4	0.04	9.2	10	4.11e-10	5.26e-8	1.10e-8
D0-unique	RNCMPT00089	SRSF10	AGAGARR	45	0.42	4	0.04	9.2	10	4.11e-10	5.26e-8	1.10e-8
D0-unique	RNCMPT00067	SRSF9	GGRWGGA	700	6.50	504	4.68	1.39	8.9	8.92e-9	1.14e-6	1.79e-7
D0-unique	RBPMap-ESRP1-1	ESRP1	GGGKGG	913	8.48	717	6.66	1.27	8.2	6.64e-7	8.50e-5	1.07e-5
D0-unique	RBPMap-FUBP1-2	FUBP1	UDUUU	1708	15.86	1447	13.44	1.18	7.5	1.82e-6	2.32e-4	2.43e-5
D0-unique	RNCMPT00044	PCBP2	CCUYCCC	256	2.38	166	1.54	1.54	9.5	6.87e-6	8.80e-4	7.50e-5

D0-unique	RNCMPT00117	HuR	UUUGUUU	579	5.38	440	4.09	1.32	9.2	7.48e-6	9.57e-4	7.50e-5
D0-unique	RNCMPT00090	SRSF10	AGAGAMA	139	1.29	76	0.71	1.82	10	1.04e-5	1.33e-3	9.24e-5
D0-unique	RNCMPT00056	RBM8A	GCGCGCG	229	2.13	150	1.39	1.52	11	2.91e-5	3.72e-3	2.34e-4
D0-unique	RNCMPT00052	RBM4	GCGCGGG	150	1.39	88	0.82	1.7	13	3.52e-5	4.51e-3	2.43e-4
D0-unique	RBPMap-EIF4G2-3	EIF4G2	GGUYGC	639	5.94	504	4.68	1.27	10	3.63e-5	4.64e-3	2.43e-4
D0-unique	RBPMap-BOLL-2	BOLL	UUUDUUU	1938	18.00	1704	15.83	1.14	6.2	5.62e-5	7.19e-3	3.47e-4
D0-unique	RBPMap-EWSR1-2	EWSR1	GGGDGGGG	1261	11.71	1081	10.04	1.17	7.6	1.08e-4	1.38e-2	6.17e-4
D0-unique	RNCMPT00113	RBM4	GCGCGSG	341	3.17	251	2.33	1.36	10	1.24e-4	1.59e-2	6.64e-4
D0-unique	RNCMPT00112	HuR	UUUGUUU	495	4.60	389	3.61	1.27	9.6	2.04e-4	2.61e-2	1.02e-3
D0-unique	RBPMap-FUS-1	FUS	GGKGG	246	2.28	174	1.62	1.41	10	2.58e-4	3.31e-2	1.22e-3
D0-unique	RNCMPT00176	MSI1	UAGUWRG	2851	26.48	2596	24.11	1.1	6.5	2.89e-4	3.69e-2	1.29e-3
D0-unique	RNCMPT00032	HuR	UUWUUUU	879	8.16	742	6.89	1.18	8.8	3.63e-4	4.64e-2	1.53e-3
D0-unique	RNCMPT00036	LIN28A	HGGAGAA	712	6.61	592	5.50	1.2	8.7	4.88e-4	6.24e-2	1.96e-3
D0-unique	RNCMPT00070	SNRNP70	RWUCAAG	244	2.27	177	1.64	1.38	11	6.35e-4	8.13e-2	2.38e-3
D0-unique	RNCMPT00018	FUS	UGCGCGC	86	0.80	48	0.45	1.78	13	6.52e-4	8.34e-2	2.38e-3
D0-unique	RNCMPT00041	MSI1	UAGUWRG	2281	21.19	2071	19.24	1.1	7	7.66e-4	9.80e-2	2.67e-3
D0-unique	RNCMPT00049	RBM28	GWGUAGW	61	0.57	33	0.31	1.82	11	2.54e-3	3.25e-1	8.43e-3
D0-unique	RNCMPT00026	HNRNPK	CCAAMCC	1050	9.75	925	8.59	1.13	7.8	2.63e-3	3.36e-1	8.43e-3
D0-unique	RNCMPT00045	PPRC1	SSGCGCS	458	4.25	379	3.52	1.21	9.5	3.49e-3	4.47e-1	1.08e-2
D0-unique	RNCMPT00186	PCBP1	CCUWWCC	1543	14.33	1397	12.98	1.1	7.5	3.74e-3	4.79e-1	1.10e-2
D0-unique	RBPMap-CNOT4-1	CNOT4	ACACAG	2420	22.48	2237	20.78	1.08	7.1	3.82e-3	4.89e-1	1.10e-2

D0-unique	RNCMPT00020	FXR2	GGACRRG	3040	28.24	2838	26.36	1.07	5.5	4.37e-3	5.60e-1	1.21e-2
D0-unique	RNCMPT00043	PABPC4	AAAAAAA	402	3.73	332	3.08	1.21	8.8	5.41e-3	6.93e-1	1.45e-2
D0-unique	RNCMPT00185	KHDRBS2	RAUAAAM	2128	19.77	1967	18.27	1.08	7.1	6.20e-3	7.94e-1	1.61e-2
D0-unique	RNCMPT00136	HuR	UUGGUUU	738	6.85	645	5.99	1.14	9	6.67e-3	8.54e-1	1.63e-2
D0-unique	RNCMPT00110	SRSF1	MAGGACAV	1673	15.54	1532	14.23	1.09	7.5	6.69e-3	8.57e-1	1.63e-2
D0-unique	RNCMPT00086	ZC3H14	UUUGUUU	1812	16.83	1668	15.49	1.09	7.3	7.67e-3	9.81e-1	1.79e-2
D0-unique	RNCMPT00073	SRSF7	GACGACGR	328	3.05	268	2.49	1.22	8.9	7.79e-3	9.98e-1	1.79e-2
D0-unique	RNCMPT00050	RBM3	GAUACGA	1790	16.63	1649	15.32	1.09	6.8	8.48e-3	1.09e0	1.89e-2
D0-unique	RNCMPT00154	RBM5	GAAGGAG	461	4.28	394	3.66	1.17	9.4	1.20e-2	1.53e0	2.60e-2
D0-unique	RNCMPT00063	SAMD4A	GCUGGMC	1093	10.15	990	9.20	1.1	8.3	1.27e-2	1.63e0	2.68e-2
D0-unique	RNCMPT00083	YBX1	AACAUCA	1481	13.76	1365	12.68	1.08	6.8	1.55e-2	1.99e0	3.20e-2
D0-unique	RNCMPT00172	IGF2BP3	AMAHWCA	958	8.90	872	8.10	1.1	7.5	2.34e-2	3.00e0	4.70e-2
Shared targets	RBPMap-FUBP1-2	FUBP1	UDUUU	3364	31.90	2819	26.74	1.19	7	2.22e-12	2.84e-10	1.89e-10
Shared targets	RNCMPT00032	HuR	UUWUUUU	867	8.22	649	6.16	1.34	9.1	1.19e-8	1.52e-6	5.06e-7
Shared targets	RNCMPT00160	HNRNPH2	GGGAGGG	1736	16.46	1427	13.53	1.22	7.9	2.12e-8	2.71e-6	6.03e-7
Shared targets	RNCMPT00044	PCBP2	CCUYCCC	360	3.41	230	2.18	1.56	9.4	4.87e-8	6.23e-6	1.04e-6

Shared targets	RNCMPT00086	ZC3H14	UUUGUUU	1091	10.35	873	8.28	1.25	8.1	4.76e-7	6.09e-5	6.99e-6
Shared targets	RNCMPT00150	ESRP2	UGGGGAU	650	6.16	485	4.60	1.34	9	5.41e-7	6.92e-5	6.99e-6
Shared targets	RNCMPT00156	CNOT4	GACAGAN	63	0.60	19	0.18	3.2	12	5.73e-7	7.34e-5	6.99e-6
Shared targets	RNCMPT00117	HuR	UUUGUUU	347	3.29	234	2.22	1.48	9.5	1.58e-6	2.02e-4	1.68e-5
Shared targets	RBPMap-BOLL-2	BOLL	UUUDUUU	754	7.15	589	5.59	1.28	6.9	3.72e-6	4.77e-4	3.53e-5
Shared targets	RNCMPT00112	HuR	UUUGUUU	1745	16.55	1522	14.43	1.15	7.1	5.11e-5	6.54e-3	4.04e-4
Shared targets	RNCMPT00049	RBM28	GWGUAGW	78	0.74	36	0.34	2.14	12	5.21e-5	6.67e-3	4.04e-4
Shared targets	RNCMPT00070	SNRNP70	RWUCAAG	625	5.93	501	4.75	1.25	9.8	1.22e-4	1.56e-2	8.04e-4
Shared targets	RNCMPT00176	MSI1	UAGUWRG	2154	20.43	1919	18.20	1.12	7.3	1.22e-4	1.57e-2	8.04e-4
Shared targets	RBPMap-ELAVL4-1	ELAVL4	UAAUU	1161	11.01	998	9.47	1.16	9.5	2.43e-4	3.11e-2	1.48e-3
Shared targets	RBPMap-FUS-1	FUS	GGKGG	141	1.34	94	0.89	1.49	11	1.31e-3	1.68e-1	7.34e-3

Shared targets	RNCMPT00172	IGF2BP3	AMAHWCA	1898	18.00	1717	16.28	1.11	6.9	1.38e-3	1.76e-1	7.34e-3
Shared targets	RNCMPT00084	YBX2	AACAWCD	877	8.32	756	7.17	1.16	9.2	1.49e-3	1.90e-1	7.46e-3
Shared targets	RNCMPT00108	SRSF1	GGAGGRV	2199	20.86	2016	19.12	1.09	7.1	2.53e-3	3.23e-1	1.20e-2
Shared targets	RNCMPT00045	PPRC1	SSGCGCS	352	3.34	284	2.69	1.24	10	3.92e-3	5.02e-1	1.76e-2
Shared targets	RNCMPT00109	SRSF1	GGAGGRV	2299	21.80	2128	20.18	1.08	6.9	5.31e-3	6.79e-1	2.09e-2
Shared targets	RNCMPT00090	SRSF10	AGAGAMA	235	2.23	182	1.73	1.29	10	5.40e-3	6.91e-1	2.09e-2
Shared targets	RNCMPT00033	IGF2BP2	AMAWACA	557	5.28	475	4.50	1.17	8.4	5.83e-3	7.46e-1	2.09e-2
Shared targets	RNCMPT00021	G3BP2	AGGAURA	346	3.28	282	2.67	1.23	10	5.94e-3	7.60e-1	2.09e-2
Shared targets	RNCMPT00163	SRSF1	GGAGGAG	1987	18.84	1831	17.37	1.09	6.9	6.06e-3	7.75e-1	2.09e-2
Shared targets	RNCMPT00041	MSI1	UAGUWRG	2673	25.35	2492	23.63	1.07	6.8	6.13e-3	7.84e-1	2.09e-2
Shared targets	RNCMPT00107	SRSF1	GGAGGAN	3869	36.69	3654	34.65	1.06	4.8	6.80e-3	8.71e-1	2.23e-2

Shared targets	RNCMPT00106	SRSF1	GGAGGAM	4604	43.66	4385	41.59	1.05	4.8	1.07e-2	1.37e0	3.40e-2
Shared targets	RNCMPT00055	RBM5	GAAGGAA	840	7.97	748	7.09	1.12	8.6	1.12e-2	1.43e0	3.41e-2
Shared targets	RNCMPT00113	RBM4	GCGCGSG	526	4.99	454	4.31	1.16	8.9	1.16e-2	1.49e0	3.43e-2
Shared targets	RNCMPT00056	RBM8A	GCGCGCG	221	2.10	176	1.67	1.25	11	1.35e-2	1.73e0	3.85e-2
Shared targets	RBPMap-ESRP1-1	ESRP1	GGGKGG	204	1.93	162	1.54	1.26	9.6	1.60e-2	2.05e0	4.30e-2
Shared targets	RNCMPT00067	SRSF9	GGRWGGA	3825	36.28	3639	34.51	1.05	6	1.61e-2	2.06e0	4.30e-2

Table S11. Differential gene enrichment results from DESeq2 analysis of fRIP-Seq data at D0 (Sheet 1) and D28 (Sheet 2). See separate file.

Table S12. Integration of differential methylation results from DSS analysis of WGBS data at D0 and D7 with fRIP-Seq, IP-MS, CUT&RUN and RNA-Seq datasets. Sheet 1: Differentially methylated regions (DMRs) and their genomic annotation at D0. Sheet 2: DMRs and their genomic annotation at D7. Sheet 3: Promoter associated DMRs at D0 and D7 that overlap H3K4me/H3K27me3 CUT&RUN peaks ('CUT&RUN_Overlap' column), encode RNAs that are HNRNPU targets or proteins interacting with HNRNPU ('DMR_PPI_fRIP_Overlap' column), or show differential RNA abundance at D28 after HNRNPU silencing based on our previous bulk RNA-Seq data. The 'DMR_DEG_Overlap' column indicates whether the associated RNA is up- or downregulated, and the 'DEG_Concordant' column denotes whether the direction of RNA abundance change is concordant with the methylation change (TRUE/FALSE). See separate file.
